# Optical manipulation of a dielectric particle along polygonal closed-loop geometries within a single water droplet

**DOI:** 10.1038/s41598-021-92209-9

**Published:** 2021-06-16

**Authors:** Junbum Park, Seongjin Hong, Yong Soo Lee, Hyeonwoo Lee, Seokjin Kim, Kishan Dholakia, Kyunghwan Oh

**Affiliations:** 1 Department of Physics, Yonsei University, 50 Yonsei-ro, Seoul, 03722 Korea; 2 Center for Quantum Information, Korea Institute of Science and Technology (KIST), Seoul, 02792 Korea; 3 SUPA, School of Physics and Astronomy, University of St Andrews, North Haugh, Fife, KY16 9SS UK

**Keywords:** Optical techniques, Fluid dynamics

## Abstract

We report a new method to optically manipulate a single dielectric particle along closed-loop polygonal trajectories by crossing a suite of all-fiber Bessel-like beams within a single water droplet. Exploiting optical radiation pressure, this method demonstrates the circulation of a single polystyrene bead in both a triangular and a rectangle geometry enabling the trapped particle to undergo multiple circulations successfully. The crossing of the Bessel-like beams creates polygonal corners where the trapped particles successfully make abrupt turns with acute angles, which is a novel capability in microfluidics. This offers an optofluidic paradigm for particle transport overcoming turbulences in conventional microfluidic chips.

## Introduction

Since the first demonstration of optical tweezers by Ashkin et al.^1^ optical manipulation of dielectric particles has evolved for various interdisciplinary science and engineering applications^2–9^. To provide an appropriate optical intensity gradient, the key requirement for optical trapping, several techniques have been developed based on conventional bulk optics such as interference patterns, special lens arrays, as well as the more conventional high numerical aperture objective lens^10–12^. In recent years there has been a drive to extend these conventional optical trapping techniques further into the microfluidic domain whilst increasing the spatial range and scope of optical manipulation. As a result, various beam shaping methods^13–18^ have been reported. Amongst them, the Bessel beam has attracted a high degree of attention due to its so-called propagation invariant (“non-diffracting”) and self-healing” nature^19–21^. The Bessel beam allows tight confinement of the trapped particle in the transverse plane and extended the transport length along the longitudinal direction along the beam axis. In previous work, we demonstrated an all-fiber Bessel beam generator using Fourier transform^22–27^ and multimode interference^28,29^ which provided highly efficient trapping and transportation of dielectric particles in a microscopic aqueous environment, compatible with microfluidic manipulation.

To date, despite a burgeoning need for transport and capture over large areas in microfluidics for multiparticle analyses in a confined geometry, there is an absence of approaches to trap and transport dielectric particles in closed-loop geometries using fiber-optic methods. In contrast, conventional optical methods have attempted to tackle this issue, showing various dynamic transport of particles in a circuitous path: optical transport of dielectric particles in transverse closed loops of the incident light has been experimentally demonstrated using a spatial light modulator (SLM) by sequential light intensity control on the SLM pixel arrays, for instance^30–32^. However, the dimensions of the loops in such an approach method have been limited by the field of the view of the focusing optics to the range of a few microns. The SLM approach requires bulk optics to focus the light and project the optical images on the fluid and is thus constrained by the physical parameters of a microscope objective that will restrict the field of view and depth of operation. This casts a fundamental limitation for in-situ, in-vitro applications in microscopic environments and the use of such approaches over extended areas on a microfluidic chip or even within microdroplets. To understand interactions between living microscopic objects and chemical surroundings, it is also desirable to provide a circulating path within a microscopic aqueous volume where the target object can be flexibly transported at will in a controlled closed-loop for an arbitrary duration time. In our Bessel-like beam, the longitudinal scattering force is significantly stronger than transverse scattering forces, and therefore, the optical transport achieved by polygonal Bessel-like beam crossings typically provides much faster velocities and ease of temporal control compared to the use of single-beam holographic optical traps. This type of single-droplet particle-circulation technique may be readily applied to areas such as circulating tumor cell research and cellular pharmaceutical aging tests in a microscopic environment^33–36^.

In this study, we propose and experimentally demonstrate a novel method to realize an efficient all-fiber optofluidic circulation of a single “trapped and guided” dielectric particle within an aqueous droplet. In our embodiment, the optical routing in defined loops in the space and the time domain was achieved by a geometry of crossing all-fiber generated Bessel-like beams to create polygonal paths within the droplet. Our scheme can be extended to provide any type of polygonal closed loops by crossing Bessel beams with the appropriate addition of further fiber optics. The typical length of the side of the polygon for optical transportation can be flexibly controlled in a range 0–900 μm, dictated by the maximum “non-diffracting” length (NDL) of the fiber optic Bessel-like beam. The range of optical transportation in the closed-path that we achieve here is several orders of magnitude above those seen in previous studies^37–41^, which have been limited to the order of a few μm or less. Using these novel features, which have not been possible in the previous studies, we achieved an optical polygonal circuitry for dielectric particles in the longest transportation length in a reconfigurable arrangement of all-fiber Bessel-like beam generators, for the first time.

This present technique used all-fiber devices that each generate almost identical Bessel-like beams^28^. These are arranged to cross to form compact polygonal optical routes for dielectric particles within the single droplet. The particles were confined to the Bessel-like beam propagation axis by the optical gradient force in the radial direction and transported by radiation pressure along the beam’s axial propagation direction. At the junctions of the polygonal routes (where the Bessel-like beams crossed), the optical forces were optimized to enable the trapped particles to make abrupt changes in the direction including at an acute angle). We deem this to be a novel feature in all-optical manipulation that has not been observed on conventional microfluidic chips^42,43^. Detailed procedures for making optical closed-loops in a single water droplet and the dynamic characteristics of the particles’ motion were analyzed. Real-time in situ control of the optical power and 3-dimensional location of optical fibers are being routinely performed in present fiber optic industries including optical communication component assembly, optical sensor units, and biomedical instrumentations. Our fiber optic geometry is fully compatible with those techniques and therefore precise alignment and prompt optical power control can be easily upgraded to semi-automatic or fully-automatic control.

**Figure 1:**
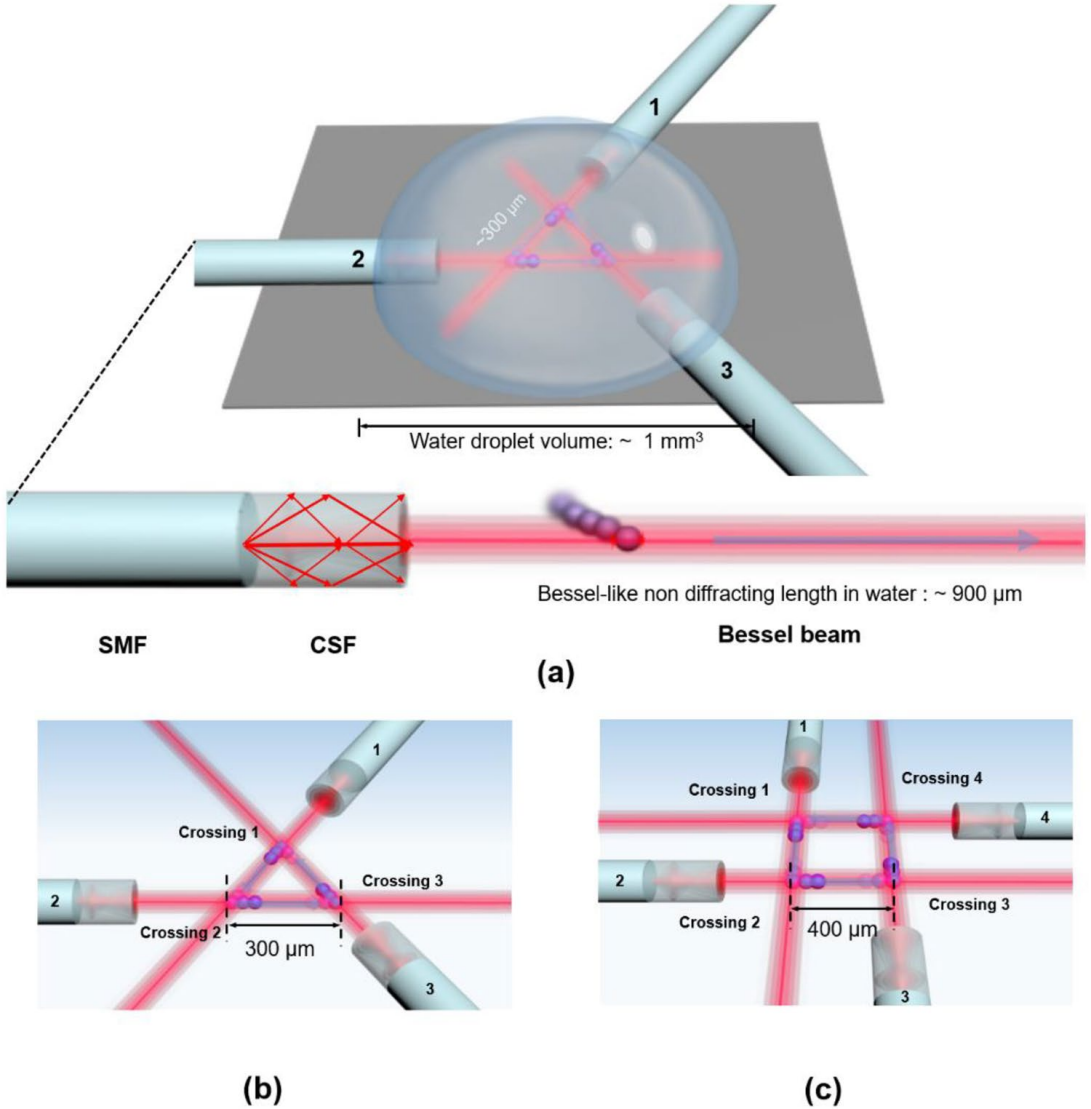
(**a**) A schematic diagram of the proposed optical polygonal circulations in a single water droplet. The lower diagram shows the multimode interference along the concatenated single mode fiber (SMF) and coreless silica fiber (CSF) to generate a Bessel-like beam, which traps and transports dielectric beads in a water droplet. (**b**) Triangular optical circulation enabled by crossing three nearly identical fiber optic Bessel-like beams in the water droplet. Here “Crossing” denotes the intersection of two Bessel-like beams. (**c**) Rectangular optical circulation enabled by crossing four nearly identical fiber optic Bessel-like beams in the water droplet.

Our proposed method is schematically shown in Fig. 1a. The fiber optic Bessel-like beam generators (BBGs) were fabricated by concatenating single mode fiber (SMF) and coreless silica fiber (CSF) where the multimode interference (MMI) produced a Bessel-like beam as described in the reference^28^. Multiple BBGs were arranged using micro-positioners to form an optical polygon within a single water droplet whose volume was ~ 1 mm^3^. Here the typical length of the central maximum of the Bessel-like beam, or its “non-diffracting” length (NDL), was measured to be approximately 900 μm in the aqueous solution. Figure 1b,c show the schematic diagrams for the triangular and rectangular optical polygonal paths, respectively. Here the sides of the optical polygons were formed along the single Bessel-like beam while the corners were formed by crossing two Bessel-like-beams at an acute angle. The length of each side was ~ 300 and ~ 400 μm for the optical triangle and the rectangle, respectively in these cases. We performed particle circulation experiments along the fabricated optical polygons within a single droplet of an aqueous solution. The motion of the microparticles was observed using a CCD camera mounted on an optical microscope, covering the whole optical polygonal path whose side length was a few hundred microns. When we confined the observation of particle motion over one side of the optical polygon, the optical microscope-CCD provided an effective spatial resolution of ~ 1 μm.

Spherical polystyrene microparticles with an average diameter of ~ 15.7 μm, similar to the size of HL-60 cells^44^, which are widely used in biomedical experiments, were suspended in deionized water. A small quantity of milk, in the order of 0.1% in volume referenced to the water solution, was also added to visualize the optical beam path by enhancing light scattering. All of the simulations and experiments were performed for a laser diode operating at a wavelength λ = 1063 nm.

## Characterization of a single fiber optic Bessel-like beam

**Figure 2:**
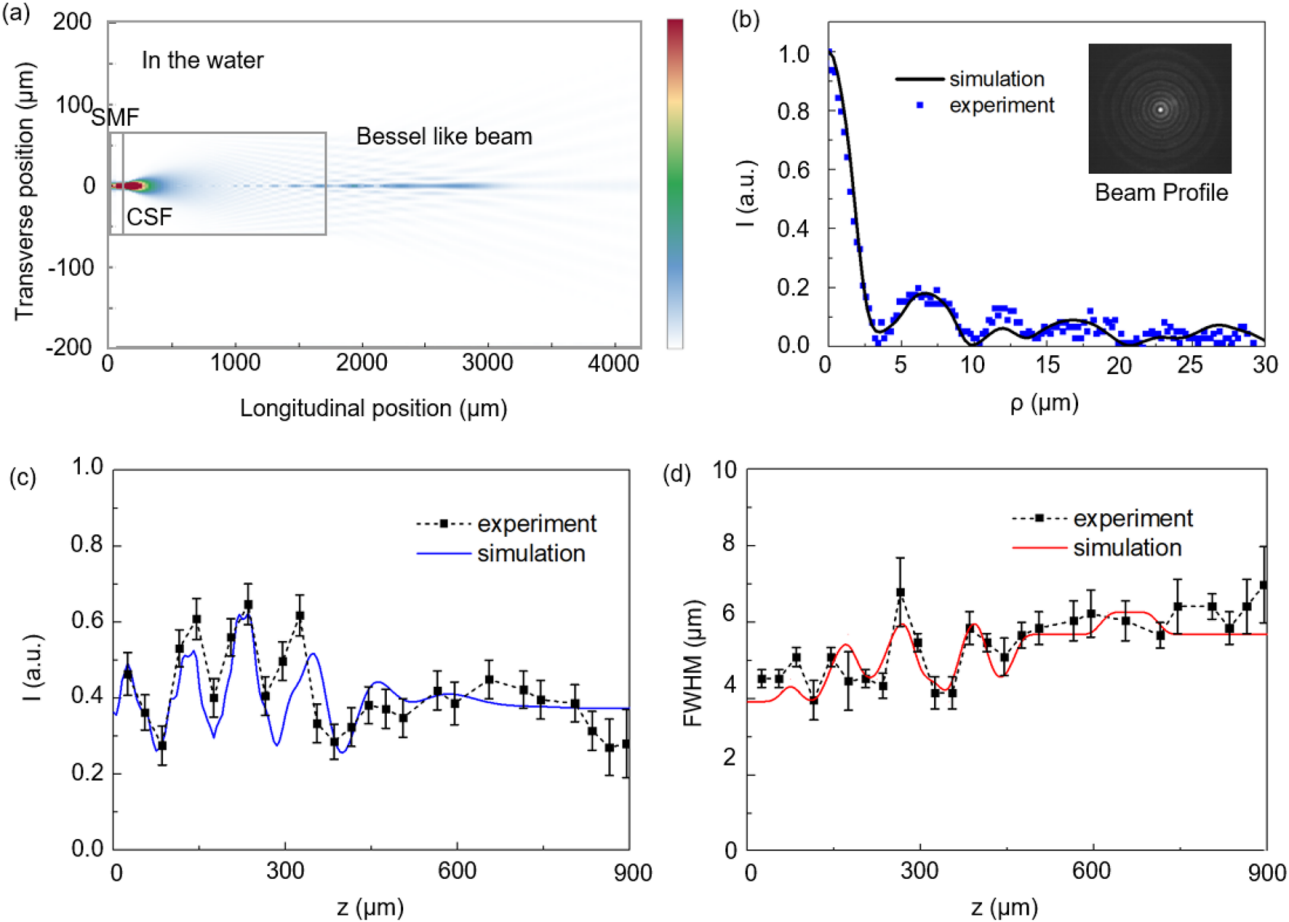
(**a**) BPM simulation of the proposed fiber-optic Bessel beam generator concatenating SMF and CSF. The axial beam intensity distribution in the water is plotted and next to the figure, its linear scale is shown in the color bar. From (**b**) to (**d**), we defined the axial position ‘z’ as the longitudinal distance measured from the end facet of CSF. (**b**) Transverse light intensity profile of the Bessel-like beam. The inset photograph is the measurements taken at z = 100 μm. (**c**) Longitudinal light intensity profile. Here we plotted the central peak intensity as a function of the axial position, z. (**d**) FWHM of the central peak as a function of z. The incident laser power was 340 mW at λ = 1063 nm.

The SMF and CSF used in the BBGs had the common outer diameter of 125 μm to provide a seamless fusion splicing. The SMF was a commercially available Corning HI1060 and CSF was made by drawing a silica glass rod in our laboratory. We optimized the CSF length to be ~ 1600 μm to generate the Bessel-like beam. We performed numerical beam propagation method (BPM) analyses, for the SMF-CSF assembly in water. The propagation invariant (“non-diffracting”) nature of the Bessel-like beam is clearly shown in Fig. 2a. To characterize the Bessel-like beam along the longitudinal direction, we defined the axial distance, ‘z’, in reference to the end facet of CSF in BBG, where z = 0. Launched laser power is defined as the output power measured at the end facet of CSF by the optical power meter in experiments. For a launched laser power of 340 mW at the wavelength λ = 1063 nm, we measured the transverse intensity profile of the beam using a CMOS image sensor at the axial distance z = 100 μm, and it showed a good agreement with the BPM simulations as summarized in Fig. 2b. The experimental image in the inset showed the typical concentric rings of *J*_*0*_*(ρ)*, while the central peak had the full width at half maximum (FWHM) of ~ 5.4 μm. The axial beam intensity distribution is shown in Fig. 2c, where the intensity of the central peak was measured as a function of z and compared with BPM results. To further quantify the NDL along the axial direction, we focused on the ratio between the central peak and the first ring in the dB scale as given in Eq. (1).

1 $$ Beam{\text{ }}ratio{\text{ }}(BR) = 10\log _{{10}} \left( {I_{{peak}} /I_{{ring}} } \right) $$

here *I*_peak_ is the light intensity at the central peak, and *I*_ring_ is the maximum intensity in the first ring of the Bessel-like beam and it is 7.9 dB for an ideal *J*_*0*_*(ρ)* beam. In our study, the NDL was defined accordingly as the axial distance, z, maintaining BR of ~ 7.9 dB. For z > NDL, the central beam fades losing the optical trapping force. In Fig. 2c, NDL was measured to be ~ 900 μm, consistent with BPM estimates. We also measured the FWHM of the central peak along the axial direction as summarized in Fig. 2d and found that the central peak maintained its FWHM of 5.4 μm ± 0.9 μm within NDL with a good agreement between experiments and BPM simulations. The difference between simulations and measurements was mainly attributed to the scattering and absorption loss in the aqueous solution to bring about the uncertainty in the beam intensity in the measurements.

**Figure 3:**
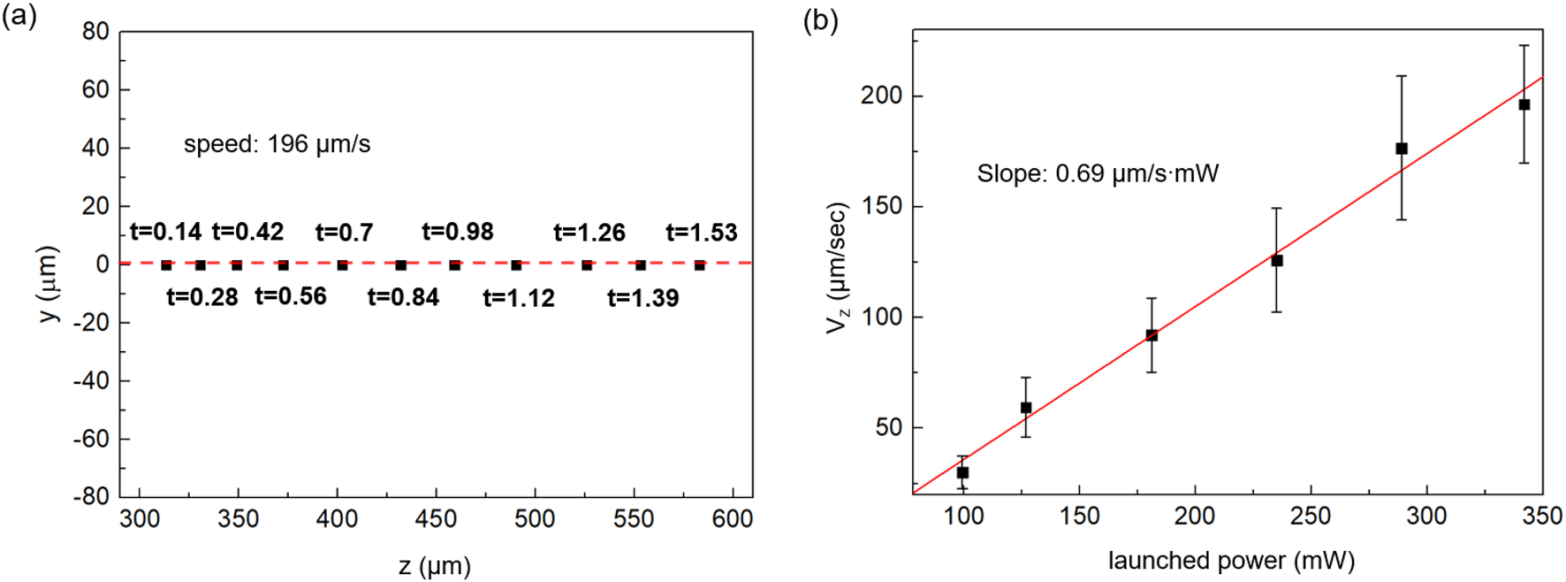
(**a**) The motion of a single polystyrene bead trapped and transported by a single Bessel-like beam in the water. The bead’s transverse position is on the y-axis while the axial position is on the z-axis. The data were taken at an elapsed time *t*. (**b**) The transportation speed along the axial direction, *V*z, as a function of launched laser power.

We further carried out trapping and transport experiments using the single Bessel-like beam in the aqueous solution and the results are summarized in Fig. 3. The transverse position, *y*, of the trapped bead, was plotted in Fig. 3a while it was transported along the axial direction. The red dashed line represents the position of the central peak of the beam and the black square is the transverse position of the trapped bead. The elapsed time was recorded for each data point. The bead was dragged toward the central peak of the Bessel-like beam near z ~ 250 μm and optically transported from z ~ 300 to ~ 600 μm. In Fig. 3a, the transverse position of the trapped particle nearly coincided with the central peak of the beam and the variation in ‘y’ position was very small within ± 1 μm. This directly indicates that the bead was highly confined by the transverse optical gradient force at the beam center, and transported in the longitudinal direction within NDL. The axial position ‘z’ versus the elapsed time was linearly fitted with an R-squared value of ~ 0.9999 and the corresponding average speed in the axial direction, *V*_*z*_, was estimated to be ~ 196 μm/s. We also used the standard finite difference method to numerically differentiate the axial distance z(t) with respect to the time, t, taking three neighboring data points^45^ and the result was consistent with the linear fitting value. We further varied the launched laser power and measured the axial speed of the trapped particle, *V*_*z*_, and the results are summarized in Fig. 3b. Within experimental errors, the axial speed, *V*_*z*_, showed a linear dependence to the launched laser power with a slope of 0.69 (μm s^−1^ mW^−1^), which enabled accurate and efficient control of particle speed in the optical polygons. We observed nonlinear thermal effects when the launched power was further increased, which will be explained in the discussion section. In order to keep the trapped particle motion in a linear regime, we restricted the launched laser power below 350 mW in the following experiments.

## Circulation of a single dielectric particle along an optical polygon

### Optical triangle

**Figure 4:**
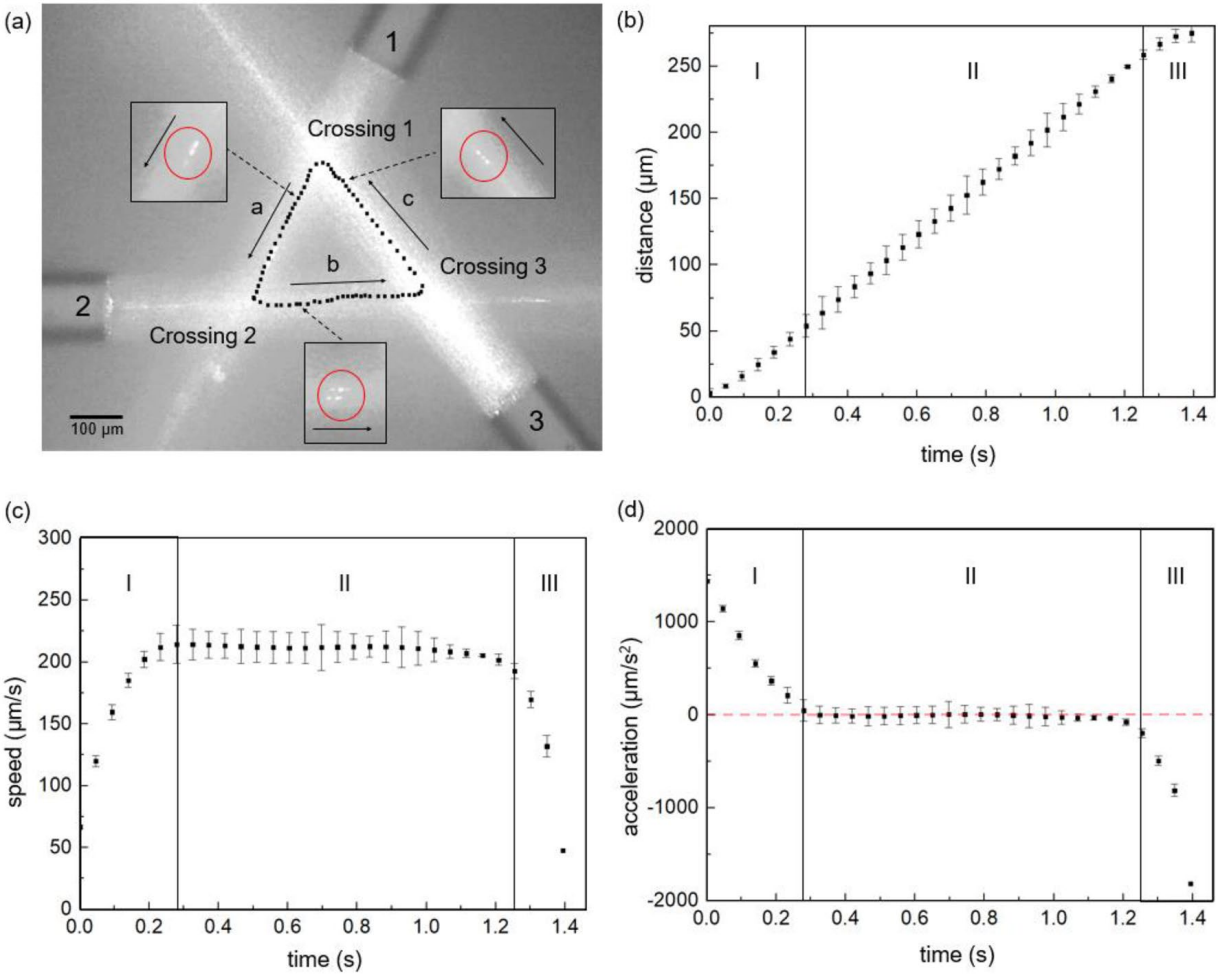
(**a**) The motion of a single polystyrene microparticle circulating along an optical triangle formed by crossing three Bessel-like beams in the aqueous solution. Three beams from BBGs labeled as 1, 2, and 3 formed a triangle (~ 60° angle at the crossings) with three sides labeled as ‘a’, ‘b’, and ‘c’. The black dots along the optical triangle represent the positions of the bead’s center measured at a constant time interval of 50 ms (**b**) The distance of the bead along the side ‘a’ as a function of time. Here the distance was measured from the Crossing 1 to the bead center. (**c**) The speed of the bead and (**d**) the acceleration in the corresponding range of (**b**). The particle motion in (**b**)–(**d**) was categorized by three segments; the segment I—‘Exit’ from the initial ‘Crossing 1’, the segment II—‘Transport’ along the side ‘a’, and the segment III—‘Entry’ to the next ‘Crossing 2.’

Confirming the transportation capability of the polystyrene bead of the single Bessel-like beam, we further fabricated multiple BBGs with nearly identical NDL of ~ 900 μm and the central peak beam FWHM of *d*_*B*_ ~ 5.4 μm similar to the BBG in Fig. 2. By aligning three BBGs in a single plane using micropositioners, we successfully made three crossings to form an optical triangle within a single water droplet. The actual images of the particle circulation along the optical triangle are overlaid as black circles in Fig. 4a and the corresponding movie files are provided in ‘Supplementary Information 1.1-optical triangle without background illumination’ and ‘Supplementary Information 1.2-optical triangle with background illumination.’ Three Bessel-like beams from BBGs labeled as 1, 2, and 3 were making ~ 60° angle at the crossings to form three sides labeled as ‘a’, ‘b’, and ‘c’. Trapping and transport of a polystyrene microparticle along three sides was accomplished by experimentally optimizing the laser power and the crossing positions. Here the power in each of the laser diodes at λ = 1063 nm in all BBGs was kept nearly the same ~ 340 mW. The side-length in the optical triangle was ~ 300 μm. Note that the particle was axially transported within the NDL of ~ 900 μm in a single BBG as shown in Fig. 2. Therefore, the unique Bessel-like beam characteristics were maintained on all sides of the optical polygons. It is noteworthy that an abrupt direction change of the trapped particle was achieved all-optically within the microscopic triangle with an area as small as ~ 3.9 × 10^–8^ m^2^, which, to date, has not been possible in conventional microfluidic chip technologies^42,43^. This was attributed to the unique crossing of Bessel-like beams and the particle dynamics along these beams, which will be explained in detail in the discussion section.

After recording the motion of a microparticle along the optical triangle using a CCD camera, we further characterized the kinematics of the trapped bead frame by frame. The black dots on the sides of the optical triangle in Fig. 4a represent the positions of the bead’s center, which were measured at a constant frame interval of 50 ms. The single loop circulation took ~ 7.11 s and it remained nearly unchanged during the repeated 10 cycles. Due to the Bessel beam crossing at an acute angle, the trapped bead showed unique dynamics that can be described in three distinctive segments: Segment I—‘Exit’ from an initial crossing; Segment II—‘Transport’ along the side, and Segment III—‘Entry’ to the next crossing. The definitions of I—‘Exit,’ II—‘Transport,’ and III—‘Entry’ are referenced with respect to the crossing of Bessel beams. These segments are shown in Fig. 4b and the bead position measured from ‘Crossing 1’ toward ‘Crossing 2’ along the side ‘a’ was plotted as a function of the elapsed time. Segment I—‘Exit’ starts from the turning point at Crossing 1 and it represents a range where the bead is under the influences of both BBG1 and BBG3. Segment II—‘Transport’ is the region where the bead experiences only the BBG1, sufficiently far from both BBG3 and BBG2. Finally, Segment III—‘Entry’ is near the next turning point at Crossing 2 where the bead is under the influence of both BBG1 and BBG2. The distance on the vertical axis of Fig. 4b was measured from Crossing 1 and the error bar represents the standard deviation of the bead position measured over ten cycles.

From the measured data, approximately one-dimensional motion z(t) of the bead along the side ‘a’, we calculated the corresponding speed and the acceleration by using the standard finite difference method to numerically differentiate the data taking three neighboring data points^45^. The results are summarized in Fig. 4c,d. The error bars in the figures correspond to the standard deviation for the data measured during ten cycles. We found similar and consistent behaviors for other sides ‘b’ and ‘c’, and therefore we focused on the motion of the particle on the side ‘a’ for further analyses.

As summarized in Fig. 4b, when the bead exited ‘Crossing 1’ its position showed a nonlinear increase in the segment I—‘Exit’. Then the bead showed a linear increase in the distance in the segment II—‘Transport’ along the side ‘a’, followed by another nonlinear behavior in the segment III—‘Entry’ as it approached the Crossing 2. The corresponding speed in Fig. 4c and the acceleration in Fig. 4d showed more clear three distinctive behaviors. The bead was accelerating in Segment I with a diminishing magnitude. In Segment II, the particle showed a characteristic linear motion with a nearly zero acceleration. In Segment III, the particle’s speed decreased with a time-varying deceleration. It was noted that in segment II—‘Transport’ the speed of the bead was consistent with the equilibrium terminal speed of the single BBG as shown in Fig. 3b. We summarized the length, speed, and acceleration of the segments I, II, and III in the optical triangle in Table 1. Here the uncertainty in the length column is due to the spatial resolution of ~ 1 μm in our measurements. The uncertainty in the speed and the accelerations represent the difference between the values calculated by the fitted line in Fig. 4b and the experimental measurements.

**Table 1:** The motion of the trapped polystyrene bead along the side ‘a’ of the optical triangle.

Segments	Length (μm)	Speed (μm s^−1^)	Acceleration (μm s^−2^)
I—‘Exit’	44 ± 1	65 ± 5 → 212 ± 5	1437 ± 7 → 101 ± 37
II—‘Transport’	215 ± 1	212 ± 12	0 ± 89
III—‘Entry’	16 ± 1	212 ± 5 → 50 ± 5	− 81 ± 40 → − 1815 ± 40

### Optical rectangle

**Figure 5:**
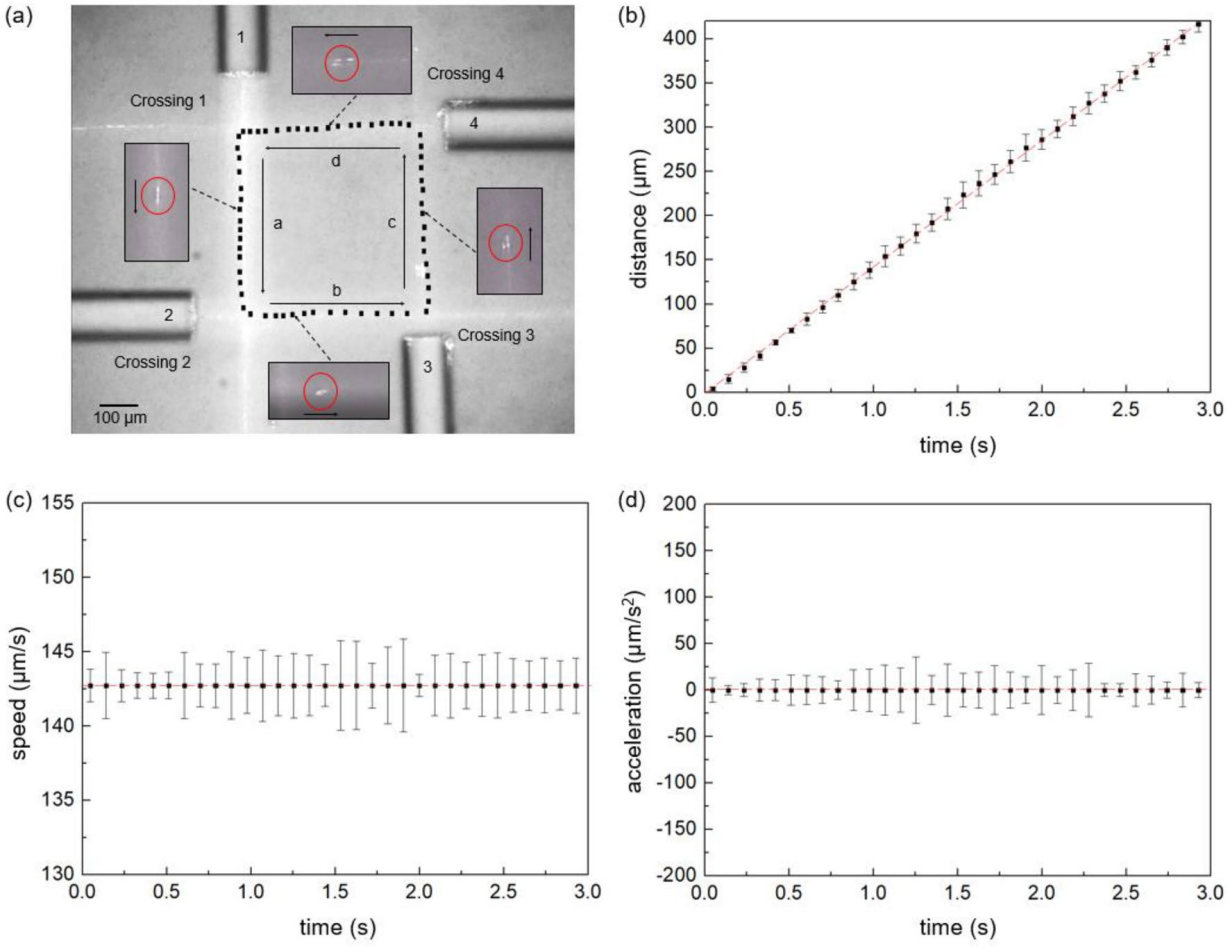
(**a**) The motion of a single polystyrene bead circulating along the sides of an optical rectangle formed by crossing four Bessel-like beams in the aqueous solution. The Bessel-like beams are labeled as 1, 2, 3, and 4 and make ~ 90° angle at the crossings to form four sides labeled as ‘a’, ‘b’, ‘c’, and ‘d’. The black dots along the optical rectangle represent the positions of the bead measured at a constant time interval of 50 ms. (**b**) The distance of the bead along the side ‘a’ as a function of time. Here the distance was measured from the Crossing 1 to the center of the bead where the bead was located between ‘Crossing 1’ and ‘Crossing 2.’ (**c**) The speed and (**d**) the acceleration of the bead as a function of time.

After confirming the complete circulation of a single particle over the optical triangle, we further proceeded to form an optical rectangle using four nearly identical BBGs and crossing them at ~ 90°. Here the power of the laser diodes in all BBGs was kept nearly the same at ~ 250 mW, which was optimized during experiments. We also accomplished complete circulations of a polystyrene bead over an optical rectangle as shown in Fig. 5a. The corresponding movie file can be found in the ‘Supplementary Information 2-optical rectangle’. The length of the side in the optical rectangle was optimized to be ~ 400 μm to ensure complete circulations of the trapped bead. Similar to the optical triangle, all the sides of the optical rectangle were within the NDL of ~ 900 μm to maintain the Bessel-like beam characteristics. The circulation area of the optical rectangle was ~ 1.6 × 10^–7^ m^2^, which is about four times larger than that of the optical triangle in Fig. 4.

We characterized the dynamics of the trapped particle and captured its motion at a constant frame interval of 50 ms. The black dot on the sides of the optical rectangle in Fig. 5a represents the position of the bead’s center, which was averaged over two frames. The single loop circulation took ~ 10.7 s and it remained nearly unchanged during the repeated three cycles. In contrast to the optical triangle, we observed a linear motion with a constant speed along all sides of the optical rectangle including the four crossings. The displacement of the trapped bead along the side ‘a’ of the optical rectangle is shown in Fig. 5b, which can be fitted to a straight line with a high correlation, R-squared value of ~ 0.99968. The slope of the linear fitting in Fig. 5b well corresponded to the average speed of the bead ~ 143 μm s^−1^ in Fig. 5c, Note that the average speed was also consistent with the equilibrium terminal speed of the single BBG as shown in Fig. 3b. We observed no significant acceleration of the bead as shown in Fig. 5d, which is attributed to no interplay for the particle between crossing beams in the optical rectangle.

## Discussion

### Scattering force and gradient force analyses

In optical manipulation of dielectric particles, the scattering force and the gradient force are the main driving forces. In our experiment, the diameter of the polystyrene bead, *d* ~ 15.7 μm, was significantly larger than the light wavelength of λ = 1.063 μm, which places our work close to the ray optics regime^46^. And therefore the scattering force along the beam axis is repulsive, pushing the particle away from the end facet of coreless silica fiber (CSF). Using a classical ray optics approach, an analytic expression for the scattering force, *F*_scatt_, is given as in Eq. (2)^46,47^.

2 $$ F_{{scatt}}  = \frac{{n_{{water}} Q_{{scatt}} P}}{c} $$

here *P* is the power of the light ray, and *Q*_scatt_ is the scattering efficiency. *n*_water_ is the refractive index of the water, and *c* is the speed of the light. Also, the bead interacts with the light by reflection and transmission at the interface. At λ = 1.063 μm the refractive indices of CSF, water, and polystyrene are 1.4563, 1.3246, and 1.5717, respectively^48–50^. In ray optics, it is also known^46^

3 $$ Q_{{scatt}}  = 1 + R\cos 2\theta  - \frac{{T^{2} \left\{ {\cos \left( {2\theta  - 2\theta _{T} } \right) + R\cos 2\theta } \right\}}}{{1 + R^{2}  + 2R\cos 2\theta _{T} }} $$

here, *θ* and *θ*_T_ are the incident angle and the angle of refraction at the polystyrene bead-water interface, respectively. *R* (the reflection coefficient), and *T* (the transmission coefficient) are also calculated at the polystyrene bead-water interface and *R* ~ 0.004, and *T* ~ 0.996 at λ = 1063 nm^17,51,52^. In the opposite direction to the scattering force, the drag force *F*_d_^53–55^ is exerted on the bead to make the trapped particle undergo the characteristic linear motion with a terminal speed as shown in Fig. 3.

4 $$ F_{d}  = 3\pi \mu _{{H_{2} O}} dv $$

Theoretical the terminal velocity, *v*_theory_, of the bead is given by

5 $$ v_{{theory}}  = \frac{{F_{{scatt}} }}{{3\pi \mu _{{H_{2} O}} d}} $$

The dynamic viscosity of water, *μ*_*H2O*_, was estimated to be ~ 0.547 × 10^–3^ N s m^−2^^56^ considering the thermal effect caused by a finite absorption of the laser in the water.

**Figure 6:**
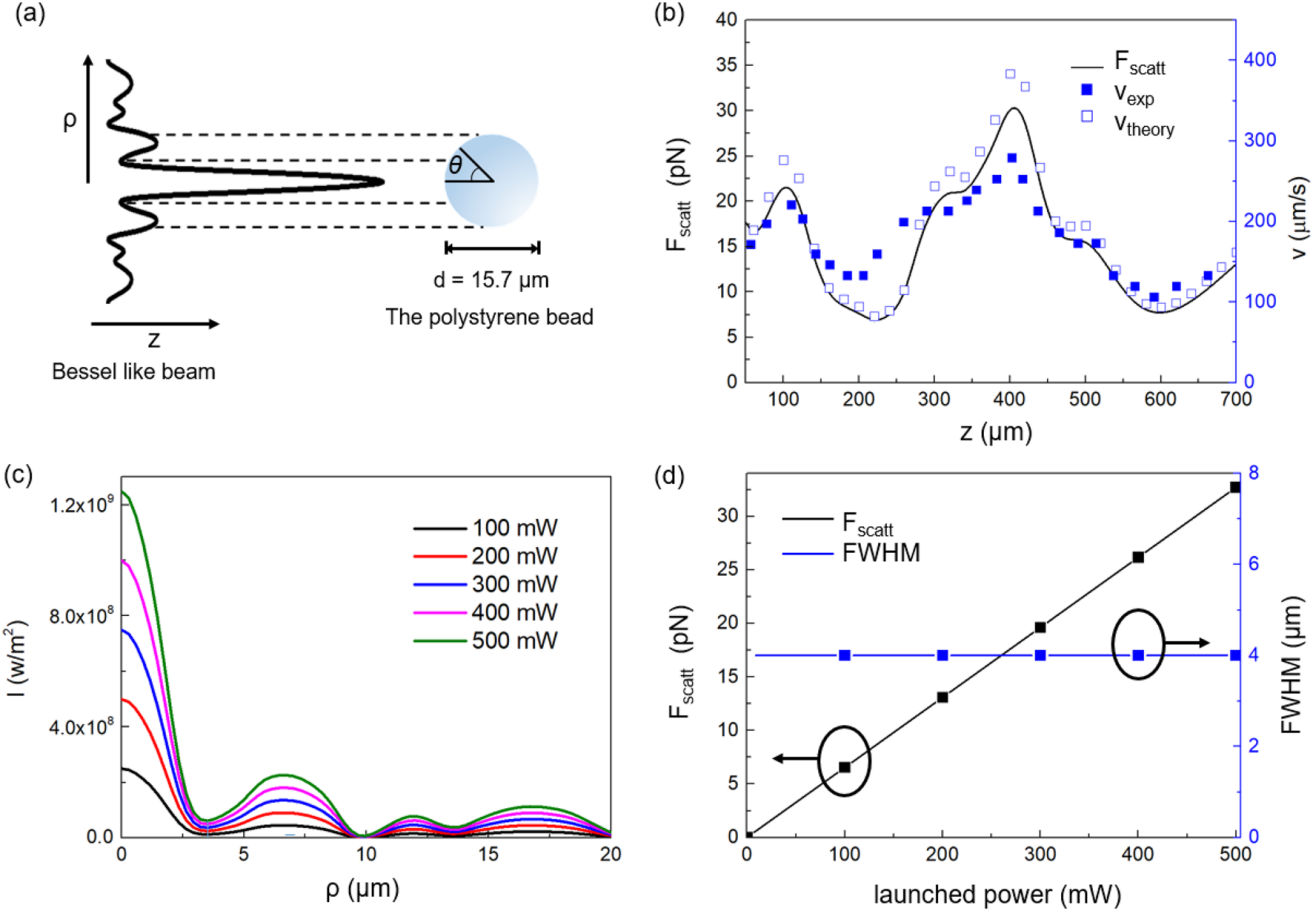
The scattering force analyses for the single fiber optic Bessel-like beam using BPM. (**a**) Relative dimension of the Bessel-like beam and the polystyrene bead. (**b**) The scattering force, *F*_scatt_, on the bead and its terminal speed as a function of the axial distance, z. The launched laser power was 342 mW. Experimentally measured speed, *v*_exp_, and *v*_theory_, estimated by BPM are compared. (**c**) The transverse intensity profile of the Bessel-like beam as a function of the radial coordinate for various launched laser powers calculated by using BPM. (**d**) *F*_scatt_ and FWHM of the central peak as a function of the launched laser power. In (**c**) and (**d**), we assumed z = 100 μm.

Similar to previously realized Bessel beams^16^, our Bessel-like beam had ~ 10% of the overall power confined to the central peak. In Fig. 6a, our Bessel-like beam and the bead are shown with a relative scale, representing FWHM of the central peak beam of ~ 5.4 μm and the bead diameter of ~ 15.7 μm. The light incident angle *θ* in Eq. (3) is shown and its range includes the central peak and the first ring of the Bessel-like beam as indicated by the horizontal dashed lines.

We also analyzed the scattering force impinging upon the polystyrene bead by our Bessel-like beam. Using Eqs. (2) and (3), and BPM simulations *F*_scatt_ was plotted in solid line as a function of the axial displacement, z, in Fig. 6b, which corresponds to the optical triangle. Here, we confined the axial distance, z, from 50 to 700 μm to simulate our optical triangle and rectangle. We found the central peak of the Bessel-like mainly contributed to the scattering force. The magnitude of *F*_scatt_ varied in the range from ~ 10 to ~ 30 pN, which is consistent with prior reports^57,58^ when scaled to our laser power. For the given *F*_scatt_, we estimated the bead velocity *v*_*theory*_ from Eq. (5) and plotted it in blank squares in Fig. 6b. In experiments, the velocity of the bead was calculated from the axial positions of the bead center in each frame, and *v*_exp_ is shown in the filled squares in Fig. 6b, which showed good agreement with *v*_theory_. Note that *F*_scatt_ had a minimum near z ~ 600 μm in Fig. 6b, and this axial position was used as a crossing position with another Bessel-like beam.

The laser power dependence was further analyzed using BPM simulations in Fig. 6c,d. It has been reported that when the laser power exceeds 550 mW, thermal effects occurred to perturb optical trapping and transport^59^. As a result, we confined the laser power range to below 500 mW. The transverse intensity profiles at z = 1000 μm are plotted for various launched laser powers in Fig. 6c. We observed that the peak intensity increased with the laser power but its FWHM was maintained at ~ 5.4 μm, as shown in blue rectangles in Fig. 6d. For a nearly constant FWHM and transverse beam profile in Fig. 6c, *F*_scatt_ linearly increased with the launched laser power as shown in the left vertical axis of Fig. 6d. In the figure, the slope of ~ 0.065 (pN mW^−1^) provided an estimation of scattering coefficient *Q*_scatt_ ~ 0.143, which is consistent with prior reports^46,47^.

**Figure 7:**
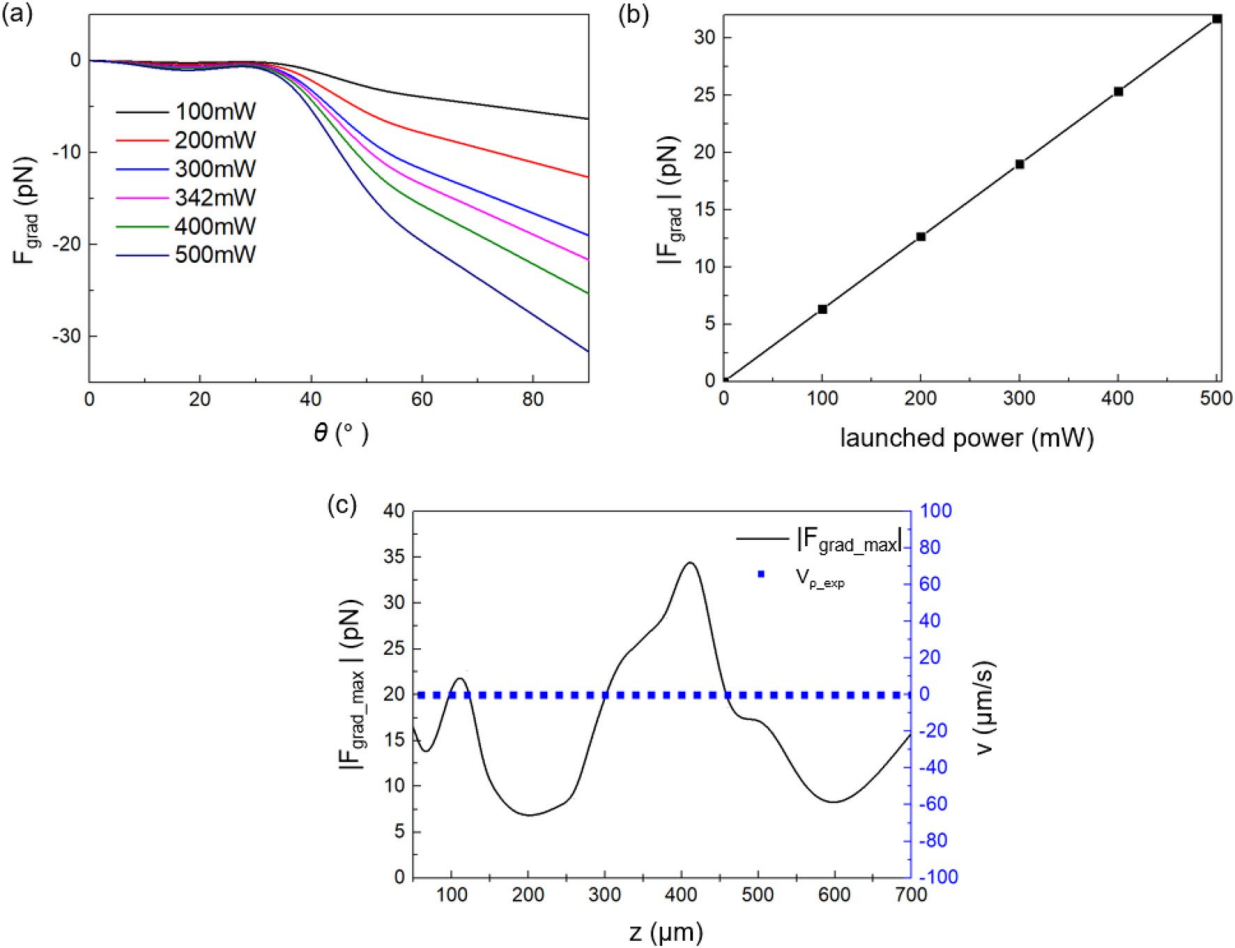
The gradient force analyses for the single fiber optic Bessel-like beam using BPM. (**a**) the gradient force, *F*_grad_, as a function of the polar position *θ* on the bead’s surface in Fig. 6a. (**b**) Magnitude of *F*_grad_max_ as a function of the launched laser power. Here we assumed z = 100 μm. (**c**) the maximum gradient force, *F*_grad_max_, is plotted as a function of z. The radial velocity, *v*_*ρ*_exp_, was also measured for the launched laser power of 342 mW.

On the transverse plane, the gradient force has the same form as in Eq. (2) but with a gradient force coefficient *Q*_grad_,

6 $$ Q_{{grad}}  = R\sin 2\theta  - \frac{{T^{2} \left\{ {\sin \left( {2\theta  - 2\theta _{T} } \right) + R\sin 2\theta } \right\}}}{{1 + R^{2}  + 2R\cos 2\theta _{T} }} $$

here, we can ignore the gravitational force which is less than 1 pN for the polystyrene bead in water. The gradient force was analyzed using BPM simulations and the results are summarized in Fig. 7. In Fig. 7a, we plotted the maximum *F*_grad_ as a function of the polar coordinate *θ* on the bead for various laser powers. Here the negative value was used for *F*_grad_ to indicate its attractive nature. We assumed the Bessel-like beam was incident through the bead center at z = 100 μm. The gradient force rapidly increased when *θ* exceeded 40°. According to the transverse intensity distribution of our Bessel-like beam as shown in Fig. 2b and 6c, the central peak’s angular position, *θ*, corresponds to 0°–40° and the polar angle range from ~ 40° to 90° corresponds to the 1’st ring on the bead’s surface. Our Bessel-like beam, therefore, can efficiently attract the bead from the 1’st ring (~ 40° < *θ* <  ~ 90°) toward the central peak (~ 0° < *θ* <  ~ 40°) by the optical gradient force from a ‘washboard potential’^60^. We also observed that *F*_grad_max_ linearly increased with the launched laser power as in Fig. 7b. Assuming ${F}_{grad\_max}={(Q}_{grad}{n}_{water}/c)P$^46^ similar to *F*_scatt_ in Eq. (2), we estimated the trapping efficiency, *Q*_grad_ ~ 0.148 from the slope in Fig. 7b. We found *Q*_grad_–*Q*_scatt_ in the single fiber optic Bessel-like beam, which efficiently enabled the optical polygonal routing as in Figs. 4 and 5.

We further estimated *F*_grad_max_ as a function of the launched laser power at the axial distance z = 100 μm as summarized in Fig. 7b. In Fig. 7c, we plotted *F*_grad_max_ as a function of the axial distance z for the launched laser power of 342 mW. The gradient force of our Bessel-like beam was estimated in the range of 6.7–35.3 pN, which is comparable to values obtained in prior bulk optics Bessel beams^60,61^. The experimentally measured radial speed of the bead, *v*_ρ_exp_, is overlaid in Fig. 7c in blue squares, whose value was kept nearly zero due to the tight trapping of the bead to the central peak of our Bessel-like beam.

### Optimization of Bessel-like beam crossings

Forming an optical polygon required delicate optimization of the crossing positions of two Bessel-like beams that served as the corners of the polygon. In experiments, the optimization was achieved by observing the particle motion using the optical microscope system while adjusting the position and laser power for each Bessel-like beam. In the optical triangle as in Fig. 4a, the crossing positions were attempted initially near z ~ 300 μm of beam 1, while z ~ 600 μm of beam 3. By placing beam 1 on the minimum *F*_scatt_ position of beam 3, the trapped particle can make an abrupt turn to switch its path to beam 1 to form a corner of the optical triangle at Crossing 1. After confirming the particle’s turn with an acute angle of ~ 60° at Crossing 1, we proceeded optimization of the crossing positions of beams 1, 2 for Crossing 2, and then beams 2, 3 for Crossing 3 in a sequential manner, to make a complete optical triangle. The main criteria of optimization were: 1) the positions of the Bessel-like beams at the crossing, and 2) the optical power of individual Bessel-like beams, which provided the desired turning of the trapped bead at Crossings. If the Bessel-like beams were crossed at positions too far from the minimum *F*_scatt_ position in the preceding beam, the bead continued its linear motion passing through the crossing without making the desired turn toward the other beam. The optical power of the beams was also optimized to provide multiple circulations of the trapped bead over the triangle. If the power is not sufficiently high, the trapped bead escaped from the optical paths due to drag forces. Conversely, thermal effects hindered optical transport for the case of using too high an optical power.

In the case of the optical rectangle in Fig. 5a, Crossing 1 was at z ~ 100 μm of beam 1, and z ~ 600 μm of beam 3, which enabled the efficient transfer of the particle from beam 3 to beam 1 with a 90° degree change in its transport direction. Other crossings were formed sequentially by optimizing the crossing positions of the Bessel-like beams and their optical power to ensure multiple circulations around the optical rectangle. Fiber-optic components such as fast variable optical attenuator and multiport optical switches are available, and a combination of these could be readily applied to create more complex optical polygon loops for particles.

### Dynamics near Bessel-like beam crossing points

**Figure 8:**
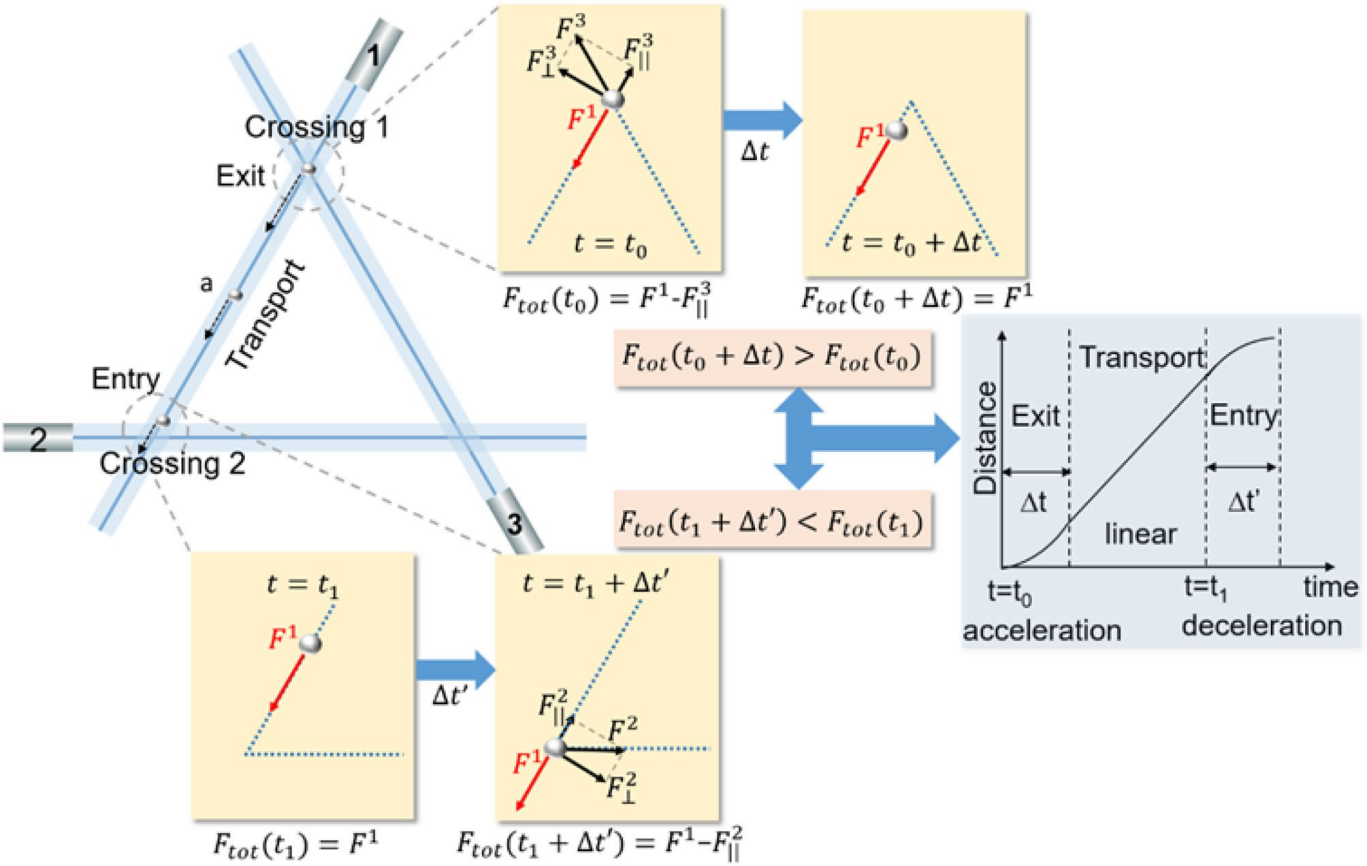
A qualitative explanation of three distinctive motions along a side of the optical triangle, ‘a’. The polystyrene bead moves from ‘Crossing 1’ to ‘Crossing 2,’ showing three segments I-Exit, II-Transport, and III-Entry.

When two Bessel-like beams cross each other at an acute angle to form an optical triangle, we observed not only the linear motion but also the acceleration and deceleration of the trapped bead in specific segments as in Fig. 4 and Table 1. Here we qualitatively explain three distinctive motions in the optical triangle as shown in Fig. 8. In the figure, the polystyrene bead moves from ‘Crossing 1’ to ‘Crossing 2’ along the side ‘a’ of the optical triangle. As the bead moves away from the Crossing 1 at time *t* = *t*_0_, both Bessel-like beams 1 and 3 are exerting scattering forces on it as indicated by *F*^1^ and *F*^3^. An expanded diagram of Crossing 1 is shown on the top right. There is a component of *F*^3^, *F*_||_^3^, parallel to the direction of *F*^1^, and the total scattering force along the side ‘a’ at *t* = *t*_0_ is given by *F*_tot_ (*t*_0_) = *F*^1^—*F*_||_^3^. After a finite time of ∆*t* in the ‘Exit’ segment as the bead escape from the influence of Bessel-like beam 3, it only experiences the scattering force *F*^1^, *F*_tot_ (*t*_0_ + ∆*t*) = *F*^1^. Since we have *F*_tot_ (*t*_0_ + ∆*t*) > *F*_tot_ (*t*_0_), the particle experiences a positive acceleration.

In the ‘Transport’ segment the particle is under the influence of only the Bessel-like beam 1 to reach a terminal speed showing a typical linear motion without an acceleration. On the contrary, as the bead further moved towards Crossing 2, the bead experiences the presence of Bessel-like beam 2. Near Crossing 2 at time *t* = *t*_1_, two Bessel-like beams 1 and 2 are exerting scattering forces on the bead as indicated by *F*^1^ and *F*^2^. See the expanded diagram of Crossing 2 on the bottom left. There is a component of *F*^2^, *F*_||_^2^, parallel to the direction of *F*^1^, and the total scattering force at *t* = *t*_1_ + ∆*t*′ is given by *F*_tot_ (*t*_1_ + ∆*t*′) = *F*^1^—*F*_||_^2^ and we have *F*_tot_ (*t*_1_ + ∆*t*′) < *F*_tot_ (*t*_1_) which results in a deceleration motion at the ‘Entry segment’.

Due to these vectorial sums of the scattering forces and the balancing drag force in the aqueous solution, we can qualitatively explain three distinctive motions on the segments as shown in the graph on the middle-right of Fig. 8, which is consistent with our experimental data in Fig. 4b.

The above features can be also attributed to the bead size (*d* ~ 15.7 μm) versus the central beam diameter (*d*_B_ ~ 5.4 μm) of our Bessel-like beam. Note that *d*_B_ is only ~ 1/3 of *d*. In Fig. 8, we drew the Bessel-like beam in a straight line, whose thickness represents the beam diameter at the central peak in reference to the polystyrene sphere. Even before the bead reaches the crossings, it is already under the influence of the other Bessel-like beam due to its large diameter. Our Bessel beam used 15.7 μm diameter particles but we can readily other particle sizes from 1 μm upwards. The exact dynamics of motion will vary with particle size but the general features shown in this present paper will be applicable. When we used other beads with *d* ~ 1 μm, we did not observe either the acceleration or the deceleration along the sides of the optical triangle in identical experimental conditions to the one presented here. Instead, we observed Brownian fluctuations along the Bessel-like beam within the central beam, indicating that the particle size versus the beam diameter plays an important role in determining the nonlinear motion along the side of the optical triangle.

In the optical rectangle, in contrast, two Bessel-like beams cross at right angles and the scattering forces from the beams are orthogonal to each other. The absence of the parallel component at the crossing resulted in a linear motion in the optical rectangle as in Fig. 5. The bead cannot experience the other Bessel-like beam’s influence until it reaches the crossing point (junction) of the optical rectangle.

It is noted that there is an angular acceleration in addition to the linear acceleration at the crossings to make abrupt turns in optical polygons. This comes about due to the interplay between beams at the crossing. A detailed description of the optical field at the Bessel beam crossing at an arbitrary angle would further require a complicated 3-D optical field calculation near the crossing for rigorous numerical analyses, which is of interest but beyond the scope of this present study.

The optical circulation continued over ~ 10 laps and we found thermal agitation of the optical path by the water absorption of the laser light could be attributed to the main cause of the circulation instability. If we choose D_2_O that has a very low absorption at the laser wavelength, instead of H_2_O, we can significantly reduce the thermal effects and can enhance the circulation efficiency and stability. A further way to reduce thermal effects would be to shift to a laser wavelength of lower absorption such as 785 nm.

Fiber optic Bessel beam generators have the same outer diameter as the conventional optical fibers so that our optical polygon scheme can be readily applied to a bio-opto-fluidic PDMS chamber, as demonstrated in Lee et al.^29^. Circulating cells in this fashion could be used to expose them to differing media and regions of complementary interrogation on chip. This is useful for disease diagnosis and analysis at the single-cell level.

## Conclusion

We experimentally achieved a directed, controlled circulation of dielectric microparticles over triangular and rectangular polygonal paths formed by crossing fiber optic Bessel-like beams within a single water droplet. The Bessel-like beams were generated by multimode interference along a concatenated single mode fiber and coreless silica fiber. An optical triangle with a side length of ~ 300 μm was obtained by crossing nearly identical three Bessel-like beams and we successfully achieved trapping and circulation of polystyrene microparticles along the sides of the optical triangle whose area was as small as ~ 3.9 × 10^–8^ m^2^. On each side of the triangle, we observed three distinctive motions characterized by the acceleration, the linear motion, and the deceleration, which was attributed to the vector sum of the scattering forces at the crossing of two Bessel-like beams and the choice of particle size with respect to the beam diameter used in this study. We also successfully demonstrated the circulation of dielectric beads around the sides of an optical rectangle (side length was ~ 400 μm). The bead was trapped and circulated over an area of ~ 1.6 × 10^−7^ m^2^. In contrast to the optical triangle, the motion of the particle along the side of the optical rectangle showed a nearly constant terminal speed of ~ 140 μm s^−1^, due to the absence of “competing” paths in circulation which occurs for all non-orthogonal geometries. This method can be readily applied to various types of dielectric particles such as cells, nano/micro particulates providing a new optical platform for bio-opto-fluidics.

## Supplementary Information

Supplementary Legends.

Supplementary Video 1.1.

Supplementary Video 1.2.

Supplementary Video 2.

## Data Availability

All data generated or analyzed during this study are included in this published article (and its Supplementary Information files).
