# Supplementary material for: Optical manipulation of a dielectric particle along polygonal closed-loop geometries within a single water droplet
Source: Sci Rep. 2021 Jun 16;11:12690. doi: 10.1038/s41598-021-92209-9 (PMC8209137; doi:10.1038/s41598-021-92209-9)
Supplement: Supplementary file 1 — Supplementary Legends. [file 41598_2021_92209_MOESM1_ESM.docx]

**Supplementary Information 1.1-optical triangle without background illumination** The circulation of the polystyrene particle along the optical triangle is recorded without background illumination in the optical microscope. The low brightness makes an automatically adjusted exposure time to result in the clear optical triangle image.

**Supplementary Information 1.2-optical triangle with background illumination** The circulation of the polystyrene particle along the optical triangle is recorded with background illumination in the microscope. The high brightness makes an automatically adjusted exposure time to result in the clear image of the trapped polystyrene bead.

**Supplementary Information 2-optical rectangle** The circulation of the polystyrene particle along the optical rectangle is recorded as the movie file without background illumination. The low brightness makes an automatically adjusted exposure time to result in a clear optical rectangle image.
